# Erosive lichen planus: an unmet disease burden

**DOI:** 10.3389/fmed.2024.1457667

**Published:** 2024-10-17

**Authors:** John H. Macken, Amal Senusi, Edel A. O’Toole, Matthew Caley, Emanuel Rognoni, Farida Fortune

**Affiliations:** ^1^Centre for Oral Immunobiology and Regenerative Medicine, Faculty of Medicine and Dentistry, Queen Mary University of London, London, United Kingdom; ^2^Department of Oral Medicine, Royal London Hospital, Barts Health NHS Trust, London, United Kingdom; ^3^Centre for Cell Biology and Cutaneous Research, The Faculty of Medicine and Dentistry, Blizard Institute, Queen Mary University of London, London, United Kingdom

**Keywords:** lichen planus, erosive, oral lichen planus, disease progression, diagnostic delay

## Abstract

**Objectives:**

To explore the demographic and clinical profile of erosive lichen planus (ELP) across multiple ethnicities within a single cohort, deepening our understanding of disease severity, progression and outcomes.

**Methods:**

A longitudinal retrospective cohort study of ELP patients in the ethnically diverse population of East London was carried out, profiling ELP (*n* = 57) against the milder reticular lichen planus (RLP) (*n* = 35).

**Results:**

A higher prevalence of ELP was observed in white populations compared to other ethnicities. Affected females were no more likely than males to develop ELP. There was an increased time to diagnosis for ELP patients (median ELP: 452 days, RLP: 312 days), spending longer in primary care before onward referral, in particular when referred by their general medical practitioner (GP) (median dentist 313 days, GP: 606 days). Depression was more likely to occur alongside ELP. Being an ex-smoker is a risk factor for ELP while being a current smoker is associated with RLP. A higher proportion of patients with ELP were missing teeth and had periodontal disease. Multisite involvement was more common in ELP, (ELP: 68% RLP: 11.43%). 55% of ELP cases developed scarring and were less likely to respond to first line medications, requiring systemic immunosuppression. The duration of follow up was increased in the ELP who were reviewed for almost twice as long as RLP patients (ELP 71 months, RLP 35 months).

**Conclusion:**

ELP takes longer to diagnose, requires prolonged tertiary care and is more resistant to treatment, when compared across multiple ethnicities. These patients have increased medical and oral health needs and are at greater risk of scarring than the reticular form. A greater education amongst primary carers on its presentation, as well as a greater understanding of the cellular and molecular mechanisms driving ELP are required to improve diagnostics and identify novel therapeutic approaches.

## 1 Introduction

Lichen planus (LP) is an inflammatory mucocutaneous condition, affecting the stratified squamous epithelia of the mucous membranes (oral mucosa, genitalia, esophagus, and conjunctiva) the skin and its associated appendages (nails, hair, and scalp) (1). Involvement of any of these sites can be isolated, concomitant, or sequential, although the skin and oral mucosa are the most frequently affected sites. Oral involvement, oral lichen planus (OLP), is estimated to affect 0.89–1.01% of the global general population, with the highest prevalence in non-Asian populations (2, 3), whereas the prevalence of cutaneous LP is less reported, with most studies focusing on its co-occurrence with oral disease (4, 5). LP most commonly affects middle-aged adults, with a higher incidence of OLP in perimenopausal females (1).

LP is classified based on its presentation and has diverse clinical variants. OLP most commonly presents with a fine network of white striae, known as reticular OLP, but similar to cutaneous disease, it may also develop papular or plaque patterns. Reticular OLP is the most common subtype and may be asymptomatic, however, pain may be triggered by factors such as spicy or acidic foods and dentifrices containing sodium lauryl sulfate (6). A more severe phenotype, known as erosive OLP, may develop. In erosive disease, white reticular lesions are accompanied by mucosal thinning, leading to ulcerative areas, which can progress to complete loss of the epithelium. Erosive LP is a variant of LP that primarily affects the oral and genital mucosa, often associated with severe pain. Other mucosal sites, such as the esophageal, nasopharyngeal, and ocular mucosa are rarely affected (1, 7). Erosive LP is a recognized subtype in both oral and genital classifications. The incidence of erosive LP is yet to be determined. LP it is thought to be a T-cell-mediated immunological disease, involving a complex interaction between immune and non-immune cells, cytokines and adhesion molecules, resulting in a dysregulated cell-mediated immune response (8), however, little is understood as to the mechanism driving the ulcerative process in erosive LP.

This study focuses on the clinical profile of erosive LP in the ethnically diverse population of east London, by detailed retrospective analysis of patients presenting to a complex mucosal disease clinic. This study aims to define the clinical pattern of erosive LP, identify possible at-risk groups, and deepen our understanding of disease progression, predictive events, complications, and outcomes.

## 2 Materials and methods

The patient archive of the complex mucosal disease clinic of the Oral Medicine department at Royal London Dental Hospital, Barts Health NHS Trust, was retrospectively reviewed over a three-month period to identify individuals with OLP.

OLP was diagnosed in accordance with the criteria endorsed by the WHO Collaborating Centre for Oral Cancer (9), which involves reaching a consensus in both clinical criteria (including the presence of bilateral, more or less symmetrical lace-like network of slightly raised white lines affecting the oral mucosa) and histopathological criteria (including the presence of a well-defined band-like predominantly lymphocytic infiltrate, confined to the superficial part of the lamina propria) (Supplementary Table 1). This correlation ensures a clear distinction from conditions with similar clinical appearances, which were excluded from this study. These include oral lichenoid contact lesions (lesions in direct association with a metal dental restoration), oral lichenoid drug reactions (lesion onset coincides with the initiation of a medication), oral graft versus host disease, oral lupus erythematosus and lichenoid lesions associated with oral submucous fibrosis (lesions associated with use of betel nut). Oral lichenoid drug reactions, secondary to systemic drugs, were excluded in accordance with the Naranjo Adverse Drug Reaction Probability Scale, which assigns a probability score to determine the likelihood of a suspected adverse drug reaction (*Total Score* ≤ 0: *Doubtful*, indicating that the reaction was likely related to factors other than the drug) (10).

Over a 3-month period, 831 patients presented to the complex mucosal disease clinic, 153 of whom had a recorded diagnosis of OLP. 53 patients were excluded as they did not satisfy both the clinical and histopathological criteria of OLP.

Patients were categorized into two groups: erosive OLP (*n* = 57), characterized by areas of erosion or ulceration, and reticular OLP (*n* = 35), defined by a lace-like network of slightly raised white lines. Due to the chronic, relapsing, and remitting nature of the disease, classification was based on the most extensive and prolonged clinical phenotype observed. A small number of isolated atrophic OLP (*n* = 8) were excluded, as this was not the focus of this research. Bullae were noted in a single case (*n* = 1) alongside a more widespread erosive phenotype and was thus classified as erosive disease. 57 patients were included in the erosive group, while 35 reticular type were included as a comparison group. Longitudinal retrospective clinical data were reviewed in all 92 cases, and were extracted by a single observer (J H Macken).

## 3 Results

### 3.1 Asian populations most affected by LP

**FIGURE 1:**
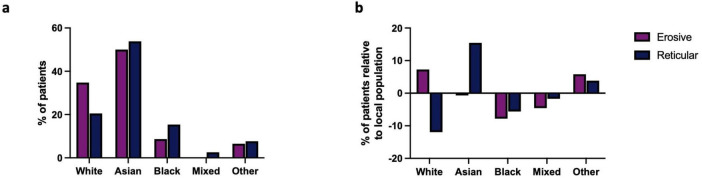
**(a)** Ethnicity of erosive and reticular LP patients in studied cohort. The Asian population was more affected by LP than any other group. **(b)** Prevalence of LP across the multiple ethnicities of the studied cohort relative to the local population: Reticular LP is more prevalent in Asians populations when compared to the local demographic. Erosive LP is more prevalent in white groups when compared to the local population.

White and Asian/British-Asian populations account for over 84.78% of the cohort studied (Figure 1a). A higher prevalence of those from Asian populations were affected by LP, irrespective of type, when compared to the local demographic of the hospital’s catchment area, and a higher proportion of erosive disease was observed in white populations compared to other ethnicities (Figure 1b). The ethnic diversity of the studied population facilitates comparison across multiple ethnic groups identifying those from Asian groups as being at increased risk of LP, with erosive disease more commonly seen in those from white backgrounds.

### 3.2 LP more common in females; females affected are no more likely than males to develop erosive LP

**FIGURE 2:**
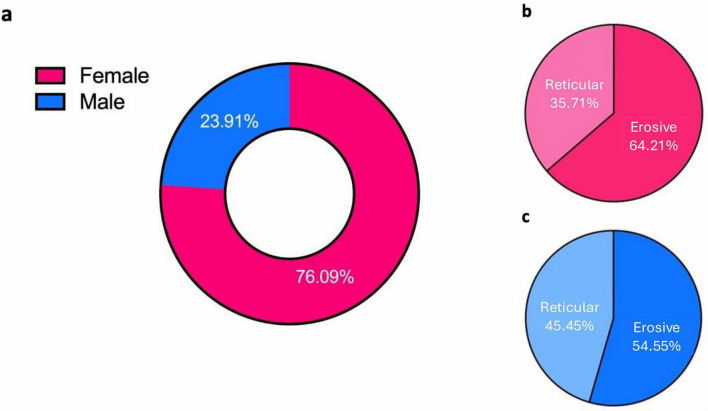
**(a)** Sex distribution within LP cohort, females (pink) vs. males (blue). **(b,c)** Sex distribution between erosive and reticular phenotypes for each sex. Dark pink and light pink represent erosive and reticular disease, respectively, in females. Dark blue and light blue represent erosive and reticular disease in males, respectively.

The patient cohort was predominately female at 76% (*n* = 70) (Figure 2a), of which 64.29% (*n* = 45) had erosive disease and 35.71% (*n* = 25) had reticular disease (Figure 2b). 54.55% (*n* = 12) of males had erosive disease and 45.45% (*n* = 10) had reticular disease (Figure 2c). There was no association between sex and LP phenotype with females affected by LP no more likely than males to develop the erosive form of the disease (Chi-squared Test *p* = 0.4117).

### 3.3 Erosive LP presents most commonly in 6th decade

**FIGURE 3:**
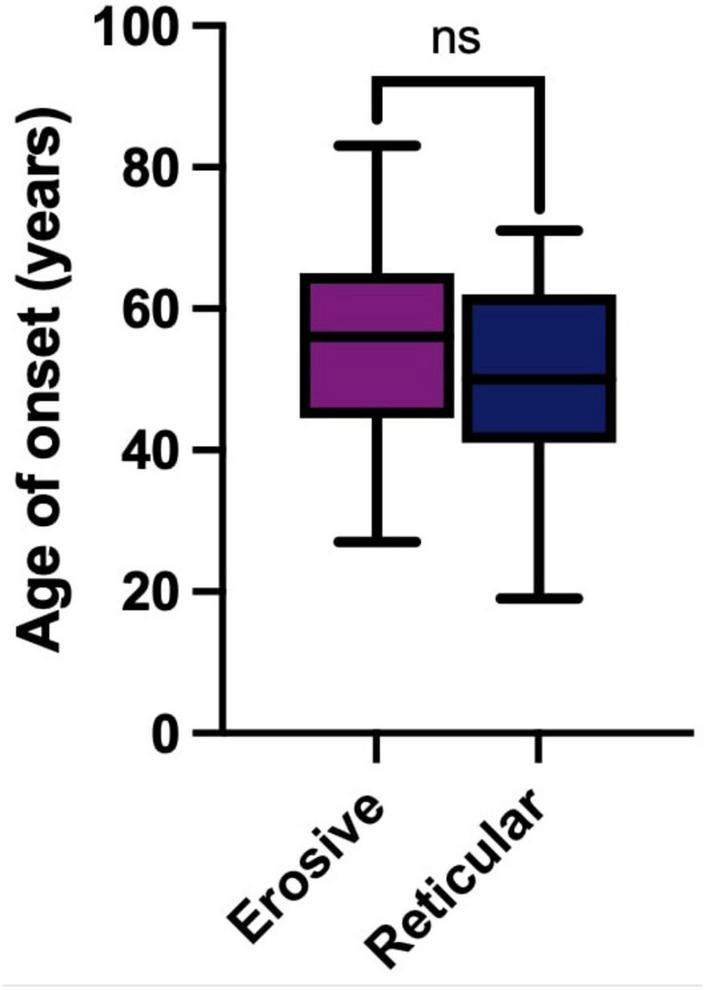
Age of onset in years of LP in the studied cohort. Erosive LP (purple) median 56 years (53–61, 95% CI). Reticular LP (blue) median 50 years (44–58, 95% CI). There is no statically significant difference (ns) between age of onset of LP in erosive or reticular phenotypes (Mann-Whitney U Test, *p* = 0.0712).

The median age of onset was 56 years (53–61, 95% CI) in erosive disease compared to a median of 50 years (44–58, 95% CI) in reticular disease. Erosive phenotype had a slightly older age of onset within the cohort, but this was not statistically significant (Figure 3). Sex had no impact on age of onset (Kruskall–Wallis Test, *p* = 0.4312, Supplementary Figure 1). This is consistent with previous studies, with LP presenting most commonly in the sixth decade of life (6).

### 3.4 Increased time to diagnosis for erosive LP

**FIGURE 4:**
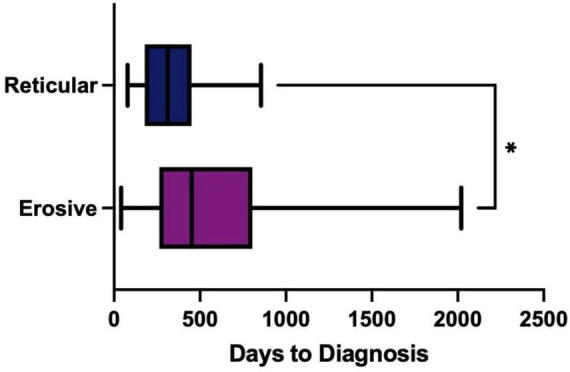
Time to Diagnosis (TTD) of erosive (purple) compared to reticular (blue) disease measured in days. Box-whisker graph where the box represents The median TTD was 452 days (311–655, 95% CI) in erosive LP (purple), compared to 312 days (198–411, 95% CI) in reticular LP (blue) using a Mann Whitney U test, *P* = 0.0289. *p < 0.05: Statistically significant.

**FIGURE 5:**
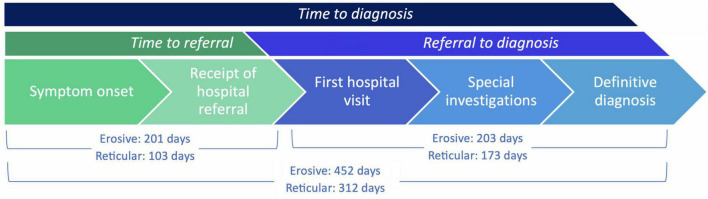
TTD (days) intervals: from *time to referral* (symptom onset to receipt of hospital referral), and from *referral to diagnosis* (receipt of hospital referral to definitive diagnosis), comparing erosive and reticular phenotypes. There was an increase in median *time to referral* for erosive LP compared to reticular LP: erosive 201 days, reticular 103 days (Mann Whitney U test, *p* = 0.0470). There was no significant difference in the median *referral to diagnosis* duration for erosive LP compared to reticular LP: erosive 203 days, reticular 172.5 days (Mann Whitney U test, *p* = 0.3549).

**FIGURE 6:**
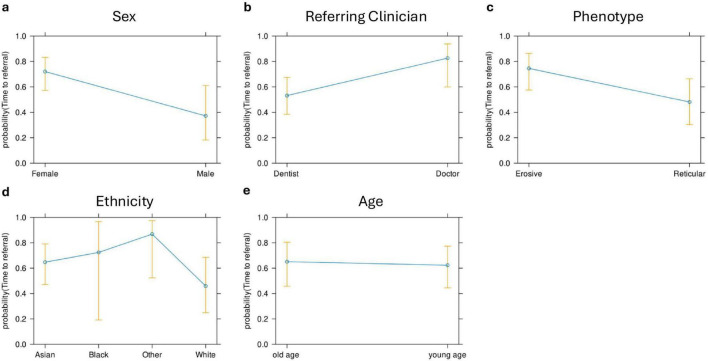
Effect plots for sex **(a)**, referring clinician **(b)**, LP phenotype **(c)**, ethnicity **(d)**, and age **(e)**. LP phenotype, sex, and referring clinician were the only predictors that had a significant effect on the *time to referral*.

The time to diagnosis (TTD) refers to the duration (number of days) between the onset of symptoms and the establishment of a definitive diagnosis by a healthcare professional. This could be determined in 71 cases. There was an increase in the TTD for erosive LP compared to reticular LP; the median TTD of erosive disease was 452 days, compared to 312 days in reticular disease (Figure 4). Logistic regression analysis shows that the patient’s age (*p* = 0.86), sex (female: male: *p* = 0.06) or ethnicity (*p* = 0.54) were not significant factors in the delay. There was however an increase in the *time to referral* (symptom onset to receipt of hospital referral) in erosive disease when compared to reticular disease; Erosive patients spent longer in primary care, a median of 201 days (118–344, 95% CI) before hospital referral, compared to 103 days (47–154, 95% CI) in reticular disease (Figure 5). A binary logistic regression models was applied to investigate *time to referral* using the following predictors: sex, age, ethnicity, LP phenotypes and referring clinician (*R*^2^ = 0.15, correct prediction 67.1%). LP phenotype had a significant impact on the independent factor (*p* = 0.025, Table 1 and Figure 6). Applying a Chi-squared test of independence for *time to referral* (≤ 3 months, > 3 months) by LP phenotypes (reticular and erosive types), erosive LP takes a longer time than reticular LP to be referred to the hospital (Chi square = 3.89, differences = 0.22, *p* = 0.048). Males were referred earlier than females in the studied cohort. Patients referred by their doctor were referred later than if they were referred by their dentist.

**TABLE 1:** Binary logistic regression analysis to investigate the *time to referral* against sex, age group (the age of the patient has been split into binary categories based on the median of the cohort age), ethnicity, referring clinician (dentist/doctor) and LP phenotype (reticular/erosive).

	Estimate	Std. error	*P*-value
Sex: male	−1.47	0.617	0.017*
Age group: young age	−0.12	0.551	0.82
Ethnicity: black	0.359	1.286	0.78
Ethnicity: other	1.28	0.957	0.181
Ethnicity: white	−0.769	0.625	0.218
Referring clinician (doctor)	1.431	0.677	0.034*
LP phenotype: reticular	−1.148	0.56	**0.04** *

Phenotype, sex, and referring clinician predictors had a significant impact on the time taken for patients to be referred to secondary care. However, age and ethnicity were identified as non-significant factors.

*p < 0.05: Statistically significant.

### 3.5 Erosive LP more commonly referred by dentists

**FIGURE 7:**
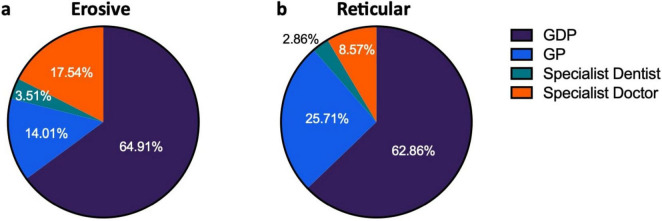
Source of referrals of patients presenting with LP, comparing erosive and reticular phenotypes. **(a)** Shows distribution of referrals for erosive patients. **(b)** Shows distribution of referral for reticular patients.

The majority of patients were referred by their dentist; 64.13% of patients were referred by their general dental practitioner (GDP), while 18.49% were referred by their general medical practitioner (GP). Patients were referred to the Oral Medicine department by dental specialists in 3.26% of cases while medical specialists were the referrer in 14.13% of cases. GDPs referred relatively equal proportions of reticular and erosive disease (erosive: 64.91%, reticular: 62.86%) while GPs referred a higher proportion of reticular disease (erosive: 14.04%, reticular: 25.71%) whereas medical specialists referred a higher proportion of erosive disease (erosive 17.54%, reticular 8.57%) (Figure 7). Patients with erosive LP were more commonly referred by their dentist, indicating the role of primary care dental services in access to care.

### 3.6 Co-morbidities and systemic medication: depression higher in erosive LP

**FIGURE 8:**
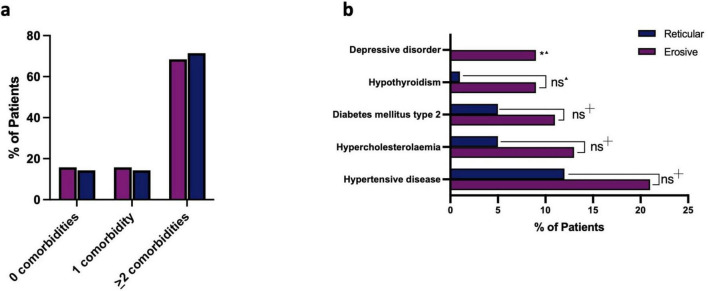
**(a)** Presence of systemic disease occurring alongside LP, comparing erosive to reticular disease. Patients with LP are more likely to present with either one comorbidity (erosive: 15.79%, reticular: 14.29%) or two or more comorbidities (erosive: 68.42%, reticular: 71.43%) than present with no comorbidities (erosive: 15.79%, reticular: 14.29%). There was no statically significant difference in the number of comorbidities between the erosive and reticular groups (Chi-squared Test *p* = 0.9547). **(b)** Breakdown of most common co-morbidities occurring alongside LP, comparing erosive to reticular disease. No statistically significant difference (ns) was reported in those affected by hypertensive disease, hypercholesterolemia, type 2 diabetes or hypothyroidism, when comparing erosive and reticular phenotypes, however, there was a statistically difference in depressive disorder, which was more likely to occur alongside erosive disease than reticular. *p < 0.05: Statistically significant. 
Fisher’s exact test. 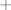
Chi-squared test.

Among the patients with LP, there was a notable prevalence of concurrent systemic diseases. There was no statically significant difference in the number of comorbidities between the erosive and reticular groups, indicating that erosive patients are no more likely to experience systemic ill-health than those with reticular disease (Figure 8a). Previous studies have explored possible associations with various systemic diseases and LP (11, 12), and within our cohort hypertension, hypercholesterolemia, type 2 diabetes and hypothyroidism were the most common comorbidities existing alongside LP. There was no statistically significant difference in those affected by these specific systemic diseases in the erosive and reticular groups, however, there was a statistical difference in depressive disorder, which was more likely to occur alongside erosive disease than reticular (Figure 8b). The patients with erosive LP were more likely to experience depression, although it is undetermined if this pre-exists the diagnosis of erosive disease or as a consequence of its development. Patient’s regular medications were also analyzed. Drugs with a reported association with an oral lichenoid drug reaction were more commonly taken by patients with erosive disease (47.37%) compared to reticular disease (31.43%), although this was not statistically significant (Chi-squared test, *p* = 0.1905, Supplementary Figure 2). No patients reported the onset of symptoms with the initiation of a medication, and oral lichenoid drug reaction was excluded in accordance with Adverse Drug Reaction Probability Scale score of ≤ 0 (10).

### 3.7 Ex-smoker status is a risk factor for erosive LP

**FIGURE 9:**
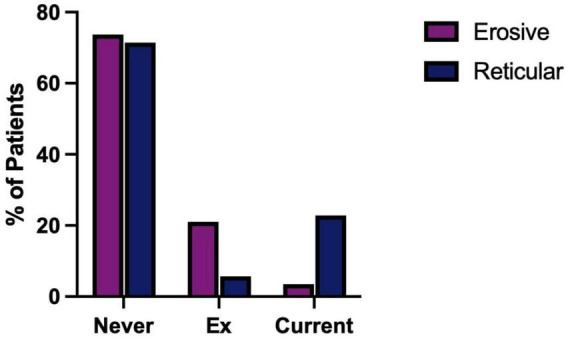
Smoking status of patients in the studied cohort. Erosive LP patients: never smoked 73.68%, ex-smokers: 21.05%, current smokers: 3.51%. Reticular LP: never smoked 71.43%, current smokers: 5.71%, current smokers: 22.86%. Chi-squared test (*P* = 0. 0046).

Patient data were analyzed to identify possible associations between erosive LP and social risk factors including smoking, alcohol consumption and smokeless tobacco and paan use. Alcohol consumption and the use of smokeless tobacco showed no significant difference between erosive and reticular groups (Supplementary Figure 3). However, the patients’ previous smoking was significant. Being an ex-smoker is associated with erosive disease while being a current smoker is associated with reticular disease, Figure 9. ANOVA test and logistic regression analysis confirmed smoking was the main risk factors that showed significant difference between erosive and reticular LP (*p* = 0.031*), meaning being an ex-smoker is a risk factor for erosive LP.

### 3.8 Erosive LP shows increased multisite involvement and scarring

**FIGURE 10:**
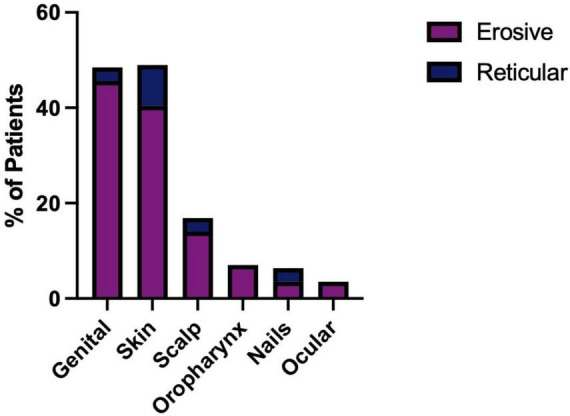
Body site involvement in erosive and reticular LP occurring alongside oral disease, including involvement of the genital mucosa, skin, scalp, oropharyngeal mucosa, nails and ocular mucosa. Erosive patients develop a more systemic phenotype with an increased prevalence of genital (45.61%), skin (40.40%) involvement compared to reticular LP. Involvement of the oropharyngeal (7.02%) and ocular mucosa (3.51%) was only observed in erosive LP.

**FIGURE 11:**
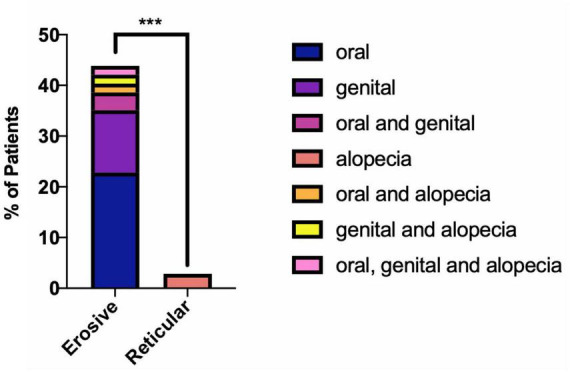
Presence of scarring in erosive compared to reticular LP. Scarring is more likely to occur alongside erosive disease (Fisher’s exact test, ***p < 0.0001). 45.61% of patients with erosive LP developed oral and/or genital scarring. No reticular LP patients developed oral or genital scarring. A single incidence of scarring alopecia was recorded in reticular oral disease (2.86%).

Patients with erosive disease are more likely to develop multisite involvement when compared to the reticular group (Supplementary Figure 4), with increased involvement of the genital mucosa, skin, scalp, nails, and ocular and pharyngeal mucosal sites (Figure 10). The genital mucosa is the most commonly affected extraoral site in erosive disease, seen in 45.61% of erosive cases, whereas cutaneous involvement was the most commonly affected site to occur alongside oral involvement in the reticular phenotype, seen in 8.57% of reticular cases. The most commonly affected oral site was the buccal mucosa (93.48% of all cases). The palate was rarely affected; 8.7% of cases, all of which were erosive disease and there was no recorded palatal involvement in reticular disease. Gingival involvement was noted in 71.74% of patients (80.70% of erosive cases and 57.14% of reticular cases). LP patients may develop scarring at the site of disease activity. Scarring developed in 27 patients, all of whom had erosive disease, except for a single case of scarring alopecia in a patient with reticular disease. The risk of scarring is significantly higher in erosive disease compared to reticular disease, where areas of ulceration and erosion are more likely to heal with scar formation (Fisher’s exact test, *p* < 0.0001, Figure 11).

### 3.9 Erosive LP is associated with compromised oral health

**FIGURE 12:**
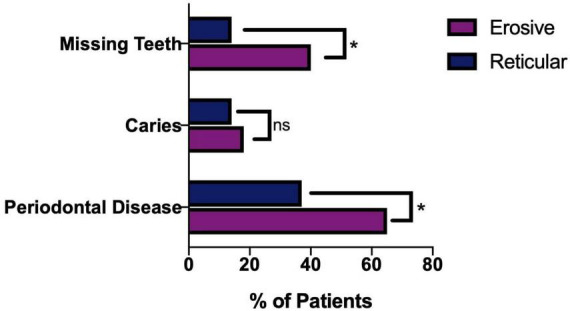
Oral health of LP patients, comparing erosive and reticular phenotypes. Patients with erosive disease are more likely to have missing teeth and periodontal disease (Fishers exact test *p* = 0.0109). There is no statically significant difference (ns) in presence of dental caries in the erosive and reticular groups (Fisher’s exact test, *p* = 0.7771). *p < 0.05: Statistically significant.

Dental records were analyzed to determine the possible impact LP has on patients’ ability to maintain their oral health. A higher proportion of patients with erosive disease were either partially dentate or edentulous, 40.35%, compared to only 14.29% of reticular patients. There was a higher prevalence of dental caries in the erosive group (17.54%) compared to the reticular group (14.29%), as well as a higher prevalence of periodontal disease 64.91% in erosive disease compared to 37.14% in the reticular group (Figure 12). This suggests that patients with erosive LP have poorer oral health related outcomes, highlighting the need for greater emphasis on oral health promotion and disease prevention programs within this cohort.

### 3.10 Erosive LP shows increased resistance to treatment and requires prolonged follow-up

**FIGURE 13:**
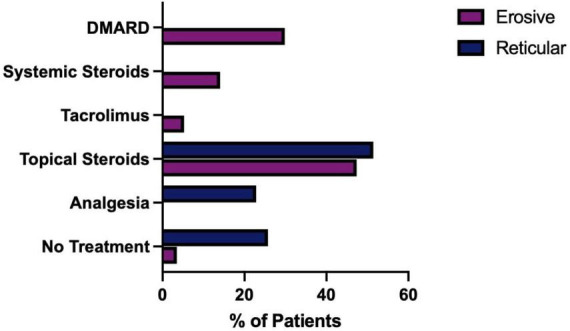
Medications used to treat, erosive compared to reticular LP. Erosive patients who had no recorded treatment were confirmed to be in disease remission.

**FIGURE 14:**
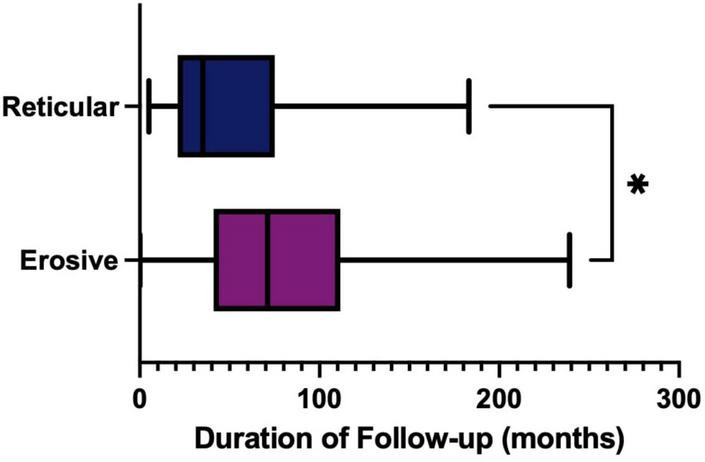
Duration of follow-up (months) in tertiary care, comparing erosive (purple) and reticular (blue) phenotypes. Erosive follow-up median 71 months (53–86, 95% CI) compared to reticular disease median 35 months (28–60, 95% CI) (*p* = 0.0155, Mann Whitney U Test). *p < 0.05: Statistically significant.

The treatment of LP is dependent on symptom severity, where asymptomatic disease may require no therapeutic intervention. First line treatment for symptomatic disease is topical corticosteroids, with topical analgesics often used as an adjunct to manage pain. In the study cohort, the reticular form of LP was more likely to require either no treatment or topical analgesics alone than the erosive form (Fisher’s exact test, *P* ≤ 0.0001). Erosive disease was more likely to fail first line therapy and require systemic medication and immunosuppression (Figure 13). As well as the increase in use of more potent and systemic medications, recalcitrant disease also requires prolonged management in tertiary care. The median duration of follow up was significantly increased in the erosive cases (71 months; 53–86, 95% CI) compared to the reticular disease (35 months; 28–60, 95% CI), Figure 14.

## 4 Discussion

Erosive LP is a clinical variant of LP, that typically presents as irregularly shaped erosions and ulceration of the mucosal tissue, with the oral and genital tissue most commonly affected. Concurrent cutaneous lesions may also be present. Ulcerated lesions may heal with scar formation, and the prevalence of scarring in erosive disease is unknown.

To date, retrospective studies on LP have been broad, higher level epidemiological studies. This study is the first of its kind focusing on erosive disease with detailed longitudinal clinical data, to understand the disease course of the erosive phenotype, identifying those at increased risk and deepening our understanding of disease progression, complications, and outcomes.

The studied population of east London is unique in its ethnic diversity, with a high proportion of white, Asian and black ethnicities. Previous studies are limited to single ethnic groups, (13–15) which clearly introduces variability with regards to standards and access to care. Our study facilitates the comparison between multiple ethnic groups in a single cohort, within a single healthcare system. The studied cohort are diagnosed and managed as part of a highly specialized service, with expertise in orogenital mucosal disease, and receive input from Dermatology and Otolaryngology as required. This ensures that accurate assessment and diagnosis can be made of Erosive LP, which frequently presents with multisite involvement. Within our cohort, a higher prevalence of those from Asian populations were affected by OLP overall, when compared to the local demographic (Figure 1). This is in contrast to two recent systematic reviews, who reported a higher prevalence in non-Asian countries (2, 3). When we compare both ethnicities in a single cohort, we see the opposite trend, possible differences in access to care or the diagnostic process could be contributory. Those from Asian backgrounds had a higher proportion of reticular disease while a higher proportion of erosive disease was observed in white populations compared to other ethnicities.

The classification of LP is flawed, with each affected body site often described as separate entities. OLP is traditionally classified into six types (reticular, plaque, papular, atrophic, erosive, and bullous) but in reality, often manifests as a mix of these types–white reticular lesions, sometimes accompanied by erythema, erosions, or bullae. Diagnostic challenges may arise when distinguishing OLP from other oral lichenoid lesions (16). Genital LP is similarly classified into classic/papulosquamous, hypertrophic, and erosive types. Erosive LP is a recognized subtype in both oral and genital classifications. There is a growing consensus for adopting a multidisciplinary approach to patient care, moving beyond the two-tier classification of oral and genital lichen planus (17). Experts in the field have emphasized the need for future cohort studies to adopt reliable, clearly defined diagnostic criteria to prevent the inclusion of other diseases with indistinguishable clinical presentations, such as other lichenoid lesions (2). To address this and ensure rigor in this study, we employed strict diagnostic criteria for Oral LP as the basis for our inclusion criteria. This rigorous approach is a significant strength of our study compared to other epidemiological studies focused on OLP. The cohort studied had confirmed clinical and histopathological findings consistent with LP, aligning with the diagnostic criteria endorsed by the WHO Collaborating Centre for Oral Cancer (9) (Supplementary Table 1) and the American Academy of Oral and Maxillofacial Pathology, which specifies that lesions are not localized to the sites of smokeless tobacco placement, not adjacent to and in contact with dental restorations, and do not correlate with the start of a medication or with the use of cinnamon-containing products (18). However, the challenge remains that LP lacks uniform diagnostic criteria across different body sites. The authors advocate for a holistic classification that recognizes mucosal lichen planus as part of a systemic disease requiring comprehensive care. In our study, LP refers to patients diagnosed with OLP and possibly other affected body sites. Reticular LP pertains to individuals diagnosed with reticular OLP, with possible involvement of other body sites. Erosive LP describes patients with erosive OLP (characterized by erosive features that are evident clinically and may also be confirmed histopathologically) and possibly other affected areas, most commonly the genital mucosa. Our cohort confirms the higher prevalence of LP in perimenopausal females, in keeping with previous studies (2).

This is the first study which focuses on the time to diagnosis of LP. Patient delays include factors such as delay in initial assessment, where patients with oral and genital symptoms delay seeking medical consultation due to social stigma and feelings of shame and embarrassment. Professional delays include proper diagnosis delay, such as repeat follow up visits without a diagnosis, and treatment for sexually transmitted infections in those with orogenital symptoms (19, 20). Oral diseases, such as OLP, often face significant diagnostic delays when isolated, exacerbated by multiple consultations and misdiagnoses (21). For instance, erosive OLP may be mistaken for oral manifestations of vesiculobullous conditions, which also present with mucosal erosions, such as mucous membrane pemphigoid (22) or pemphigus vulgaris, which has been reported to experience significant diagnostic delays when presenting with isolated oral involvement (23). Erosive disease takes longer to diagnose when compared to reticular disease, despite the erosive form typically being a more symptomatic condition. This may be impacting disease progression and is likely to be contributing to the negative impact on patients’ quality of life. There was an increased interval of symptoms to hospital registration from general medical practitioners (GP’s) compared to general dentists. Although this was not statistically significant, it does suggest a need for greater awareness and education with respect to the diagnosis of erosive disease amongst primary carers, to improve the time to diagnosis. Clinicians must have a high index of suspicion when patients present with mucosal disease and consider erosive LP among the differential diagnosis.

The general health of these patients was analyzed, to explore any health inequalities within the cohort, Figure 5. A recent systematic review and meta-analysis (24) showed a strong association between LP and depression and anxiety (20%) but failed to specify the type of LP. Our cohort showed a statistically significant difference in depression, which was more likely to occur alongside erosive disease than reticular, Figure 6. The association between smoking and LP has been reported (25), however, within our cohort, ANOVA test and logistic regression has shown that being an ex-smoker is a risk factor for erosive disease. This raises some interesting points: whether patients stop smoking due to increased pain, increased awareness and concerns regarding the premalignant potential, or if smoking cessation initiates erosive disease activity in a similar manner to other mucosal inflammatory conditions, such as ulcerative colitis (26). Nicotine reduces proinflammatory cytokines such as IL-1β and TNF-α, and alters immune cell activity by increasing suppressor CD8+ T cells and lowering the CD4/CD8 T cell ratio, which may contribute to its protective effects (27). Alrashdan et al. (28) found that smoking significantly reduces the expression of CD68+ macrophages in oral lichenoid lesions, which could affect immune surveillance. After smoking cessation, these immune-modulating effects of nicotine are lost, potentially leading to increased inflammation and the initiation of conditions like LP.

Erosive patients have poorer oral health, with a higher prevalence of tooth loss and periodontal disease. The association between periodontal disease with OLP has been supported by a recent systematic review (29). Within our cohort, patients with erosive LP were more likely to experience periodontal disease compared to reticular LP. The association between periodontal disease and erosive LP, and the complex interaction needs to be further explored. At a population level, a greater emphasis on oral health promotion and a periodontal disease prevention programme is clearly required in this cohort, and the role of general dental practitioners in the maintenance of periodontal health must be prioritized.

The association with LP and cancer progression is often discussed, and erosive disease is associated with a higher prevalence of OSCC progression compared to the reticular form (30, 31), however, this was not observed in our cohort, possibly due to the limited size of the population studied and an average follow up period of 7 years.

Erosive patients develop a more systemic phenotype with increased multisite involvement. LP has a major impact on quality of life of patients, in particular those with genital disease and those who have multisite involvement (32). We observed a higher prevalence of genital involvement than other studies (1). Our cohort are managed within highly specialized services with expertise in orogenital mucosal diseases. Comprehensive assessments of both oral and genital mucosa are conducted, possibly contributing to the higher reported prevalence. Additionally, experts in genital mucosal disease have noted that it often remains undiagnosed in many patients, potentially leading to widespread underreporting (19, 20). Erosive disease is more at risk of scarring, which can have a devastating impact on patients’ quality of life (32). Depending on the clinical presentation, scarring may result in scarring alopecia, pterygium formation and nail loss, scarring keratoconjunctivitis, vaginal stenosis, and reduced mouth opening, impacting mastication and speaking (33, 34). Ocular involvement is rare, observed in a single case within our cohort, however, the risk of irreversible damage of the visual function is significant and thus early diagnosis and intervention is paramount (7). The presence of scarring appears to be more prevalent with oral and genital disease (34) but there is a lack of understanding as to the mechanisms underpinning scar formation in Erosive LP. The longer the disease goes undiagnosed or untreated the more irreversible damage that occurs (19). As such, a multidisciplinary approach is central to accurate assessment and successful, timely diagnosis of Erosive LP, to reduce the possible sequelae of scarring.

Erosive disease is often more resistant to treatment compared to reticular disease (35). Patients are less likely to respond to first line medications, such as a single topical steroid. Recalcitrant disease may require the use of calcineurin inhibitor such as tacrolimus, or systemic immunosuppression, including systemic steroids and/or disease-modifying anti-rheumatic drugs (DMARDS), such as hydroxychloroquine, azathioprine and/or mycophenolate mofetil (36, 37). Patients with erosive disease more frequently require systemic immunosuppression, such as a DMARD, or a multi treatment approach, in an attempt to control their disease activity. A greater understanding of the cellular and molecular drivers in the erosive phenotype is required to develop targeted therapies for erosive disease. The duration of follow up was significantly increased in the erosive cases compared to the reticular disease. Erosive patients are being reviewed for almost twice as long as reticular patients. This highlights the challenges associated with treating these cases and the resulting increased burden on tertiary care, as well as the associated increased cost. Clearly further research to enhance our understanding of the drivers in erosive disease could have wider benefits to the health service.

## 5 Conclusion

In conclusion, erosive LP is a disease which crosses ethnic boundaries. Erosive LP takes longer to diagnose than the reticular form, therefore greater education amongst the public and primary carers on the presentation of this disease, as well as improved diagnostics are required. This patient group has increased needs with regards to their general and oral health, raising important questions, such as the benefits of screening for the presence of depression and anxiety symptoms, or how do we increase oral health promotion and periodontal disease prevention program in this cohort. Erosive disease is more resistant to treatment and currently, treatment developed has stalled. This is likely to have further implications, such as increased long-term complications including scarring, and patients requiring prolonged tertiary care, which increases the burden on health systems. Future research should focus on exploring the underlying drivers of erosive LP as a multisystemic disease. This could uncover novel therapeutic targets, minimize long-term complications, and provide valuable insights into the broader systemic implications of the condition, ultimately improving overall patient outcomes.

## Data Availability

The raw data supporting the conclusions of this article will be made available by the authors, without undue reservation.
