# Supplementary material for: Erosive lichen planus: an unmet disease burden
Source: Front Med (Lausanne). 2024 Oct 17;11:1457667. doi: 10.3389/fmed.2024.1457667 (PMC11524830; doi:10.3389/fmed.2024.1457667)
Supplement: Supplementary file 1 [file Data_Sheet_1.docx]

Supplementary Material

**Erosive lichen planus: an unmet disease burden**

J H Macken, A Senusi, E A O’Toole, M Caley, E Rognoni, F Fortune

| Clinical criteria | Histopathological criteria |
| --- | --- |
| - Presence of bilateral, more or less symmetrical white lesions affecting buccal mucosa, and/or tongue, and/or lip, and/or gingiva - Presence of a white papular lesions and lace-like network of slightly raised white lines (reticular, annular, or linear pattern) with or without erosions and ulcerations - Sometimes presents as desquamative gingivitis | - Presence of a well-defined band-like predominantly lymphocytic infiltrate that is confined to the superficial part of the connective tissue - Signs of vacuolar degeneration of the basal and/or supra basal cell layers with keratinocyte apoptosis - In the atrophic type, there is epithelial thinning and sometimes ulceration caused by failure of epithelial regeneration as a result of basal cell destruction. A mixed inflammatory infiltrate may be found |

Sup. table 1: Diagnostic criteria of OLP endorsed by the WHO Collaborating Centre for Oral Cancer (15)


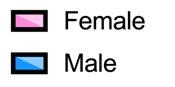


Sup. figure 1: Age of onset of LP in males and females in erosive and reticular disease. There is no statically significant difference in age on onset of LP according to sex. Kruskall-Wallis Test, p=0.4312.

Sup. figure 2: Erosive (purple) and reticular (blue) patients taking a prescribed medication with a reported association with an oral lichenoid drug reaction: erosive disease (47.37%), reticular disease (31.43%). No patients reported the onset of symptoms with the initiation of a medication.

**a b**

Sup. figure 3a: Average weekly units of alcohol consumed in the studied cohort, comparing erosive and reticular phenotypes (Chi-squared test, P= 0.0974). Sup. figure 3b: Smokeless tobacco/ paan use in the studied cohort (Chi-squared test, P= 0.1435).

Sup. figure 4: Erosive and reticular LP patients with multisite involvement other than oral mucosa (involvement of the genital mucosa, skin, scalp, oesophageal and/or oropharyngeal mucosa, nails and ocular mucosa). Fisher’s exact test (*** p<0.0001) shows that patients with erosive LP are more likely to develop multisite involvement when compared to the reticular LP. Some patients have more than one extraoral site.
